# Predicting speech-in-noise ability with static and dynamic auditory figure-ground analysis using structural equation modelling

**DOI:** 10.1098/rspb.2024.2503

**Published:** 2025-03-05

**Authors:** Xiaoxuan Guo, Ester Benzaquén, Emma Holmes, Joel Isaac Berger, Inga Charlotte Brühl, William Sedley, Steven P. Rushton, Timothy D. Griffiths

**Affiliations:** ^1^ Biosciences Institute, Newcastle University, Newcastle upon Tyne, UK; ^2^ Department of Speech Hearing and Phonetic Sciences, UCL, London, UK; ^3^ Human Brain Research Laboratory, Department of Neurosurgery, University of Iowa Hospitals and Clinics, Iowa, IA, USA; ^4^ Maastricht University, Maastricht, The Netherlands; ^5^ School of Biology, Newcastle University, Newcastle upon Tyne, UK

**Keywords:** speech-in-noise perception, auditory figure-ground, pitch perception, structural equation modelling, pure-tone audiogram, new hearing measure

## Abstract

Auditory figure-ground paradigms assess the ability to extract a foreground figure from a random background, a crucial part of central hearing. Previous studies have shown that the ability to extract static figures predicts speech-in-noise ability. In this study, we assessed both fixed and dynamic figures: the latter comprised component frequencies that vary over time like natural speech. We examined how well speech-in-noise ability (for words and sentences) could be predicted by age, peripheral hearing, static and dynamic figure-ground with 159 participants. Regression demonstrated that in addition to audiogram and age, low-frequency dynamic figure-ground accounted for an independent variance of both word- and sentence-in-noise perception, higher than the static figure-ground. The structural equation models showed that a combination of all types of figure-ground tasks and age and audiogram could explain up to 89% of the variance in speech-in-noise, and figure-ground predicted speech-in-noise with a higher effect size than the audiogram or age. Age influenced word perception in noise directly but sentence perception indirectly via effects on peripheral and central hearing. Overall, this study demonstrates that dynamic figure-ground predicts a significant variance in real-life listening better than the prototype figure-ground. The combination of figure-ground tasks predicts real-life listening better than audiogram or age.

## 1. Introduction

Tracking a target sound in a complex auditory scene is one of the core tasks that the auditory system performs and forms an important part of hearing ability. Complaints about understanding speech in noisy environments are frequently encountered in audiology clinics but are difficult to assess because the pure-tone audiogram does not fully reflect this ability [1–3]. Sentences-in-noise and word-in-noise (WiN) tests have been developed to simulate real-life speech-in-noise (SIN) situations and have been more and more used to assess real-life listening. However, responses to these tests are inevitably influenced by other factors, such as levels of education, accent and language experience as much as central sound processing. This means the current SIN test resources are difficult to generalize. For example, while SCAN-C was the most commonly used screening tool for children in UK audiology practice in 2024 [4], the US stimuli used in the test were shown to result in poorer performance and applying the US normative scores to UK children resulted in a high rate of overidentification [5]. Patients who are not able to give complex behavioural responses (e.g. repeating a sentence back or selecting sentences with a computer response matrix) also cannot be assessed with verbal SIN tests. This has motivated work to develop non-speech measures of figure-ground analysis. A non-speech auditory target-in-noise task has been developed called the stochastic figure-ground test or fixed-frequency auditory figure-ground (AFG-Fixed) [6]. The auditory figure is made of pure-tone frequency elements repeating over time and the ground is composed of similar pure-tone elements randomized over frequency and time. Modelling suggests that sound segregation is achieved based on the temporal coherence of the figure [6], requiring the auditory cortex. Brain studies implicate a network including high-level auditory cortex in humans [7,8] and in a primate model [9]. An AFG gap discrimination task was shown to predict SIN performance and explain variance in SIN independent of that explained by the pure-tone audiogram (PTA) [10].

SIN perception recruits acoustic features to better segregate sounds in noise. One of the key features is the fundamental frequency corresponding to the perception of pitch in sentences. In this study, we assess a type of AFG task in which the frequency components vary over time following the pitch contour of natural speech. This makes the stimulus more speech-like, while retaining the overall advantage of the AFG task as a ‘pure’ measure of grouping relevant to real-life listening without linguistic confounds. AFG with changing frequency patterns has been investigated with roving figures following the formants of spoken stimuli [10]. While figures generated from the first three formants of speech did not significantly predict SIN, a stimulus based on the first formant and additional components that changed over time coherently with the first formant did correlate with SIN with a small effect (*r* = 0.28). However, first-formant figure-ground was not a significant predictor in a multivariate linear regression model including PTA and the classic figure-ground. This suggested that incorporating a dynamic frequency contour into the figure-ground could potentially predict speech perception in noise, but the speech formants might not be the best frequency information to use. Another important frequency contour in speech is the fundamental frequency (*F*_0_) that determines pitch perception, which is an important basis for sound segregation [11,12]. A more primary role in grouping is suggested by work showing new-born babies track changes in pitch but not formants as reliably as adults [13]. We assess similar stimuli here with multiple frequency components that change coherently based on the pitch contours of speech sentences to examine their relationships with speech perception in noise.

Natural voiced speech contains multiple harmonics related to the fundamental frequency and is associated with pitch. Harmonicity aids hearing in noise [14]. Pitch contributes to SIN processing, especially for people with higher language or hearing competence [15–17]. In this study, we generated figures related to the harmonic structure of speech, in contrast to the non-harmonic figures used in previous work. We extracted the fundamental frequency from naturally spoken sentences and developed a new type of dynamic AFG stimulus using harmonic complexes based on this. We call this the dynamic figure-ground stimulus (AFG-Dynamic). The harmonic features make the AFG more speech-like from an acoustic perspective, without incorporating high-level linguistic cues.

We created harmonic complexes in different frequency ranges to explore the importance of the frequency range of the figure. Previous studies suggest that high-frequency hearing sensitivity based on the audiogram may be an important determinant of SIN ability [10,18,19] but have not examined complex figures in different frequency ranges. We constrained the frequency range of the AFG-Dynamic stimuli to low-frequency AFG (AFG-Low) and high-frequency AFG (AFG-High) components to explore how grouping ability in different frequency ranges contributes to SIN perception.

### (a) Predictive measures of speech-in-noise perception

The first aim of the study was to investigate whether the new dynamic AFG tests are predictive of SIN measures. We hypothesized that both versions of AFG-Dynamic tests (AFG-Low and AFG-High) can predict SIN perception and explain an extra variance of SIN independent of the PTA or the prototypical AFG-Fixed. As speech is dynamic in its frequency profile whereas single words have a relatively static frequency pattern, we predicted that the fixed-frequency AFG-Fixed better predicts word-level segregation, whereas the AFG-Dynamic tests with the changing pitch pattern better predict sentence-level sound segregation.

### (b) Modelling the relationships among auditory figure-ground perceptionage and speech-in-noise perception

The second aim of the study was to describe the relationships among the psychoacoustic measures used in the study and identify the contribution of different factors to SIN perception in a multivariate model using structural equation modelling (SEM). The current study had a complex design investigating different measures of hearing thresholds, AFG and SIN. This type of design favours the use of SEM compared with regression, as it allows having multiple observed variables indicating one latent variable (hypothetical constructs that are not directly measured but can be inferred by their observed variables) and reflects the relative importance of indirect effects, such as the interaction between covariates on outcomes. Three conceptual models based on different outcome variables were therefore constructed with assumptions on the direction of causality according to existing literature. The three outcome variables were: the WiN measure, the sentence-in-babble measure and the two measures combined. As the word- and sentence-level SIN analysis and the self-reported SIN ability tap into different domains of SIN perception, models predicting the three SIN measures separately should provide additional information on the differences in the three domains of SIN analysis when interacting with AFG, PTA and age. We also investigated the domain-general SIN by combining the three measures into one latent variable (variable 'SIN').

The fixed-frequency AFG test has been shown to predict SIN perception [10]. In this study, we added the two additional dynamic AFG measures with high and low frequencies to form an AFG latent variable that predicts SIN perception.

In terms of the exogenous variables, PTA and the participant’s age were taken into account. PTA has been shown to predict SIN ability [2,3,10,20,21]. Age has also been recognized as a key factor impacting both hearing and SIN perception [22]. Deterioration of the auditory periphery—including hair cell and cochlear nerve loss, as well as cochlear synaptopathy [23–25could all lead to decreased real-life listening ability, and these peripheral deteriorations are all tied with ageing [26]. Researchers have found a relationship between age and both AFG and SIN performance [10]. Altered auditory peripheral function could result in lowered frequency and temporal resolution, which would inevitably impact the central sound segregation ability measured by the AFG tests. However, the relationships between AFG and SIN perception were retained after accounting for age and PTA, which indicated that figure-ground measures may also index PTA- and age-independent deficits in SIN perception. Thus, we hypothesized that PTA and age would predict both SIN and AFG with age impacting PTA, and that AFG can independently predict SIN after accounting for age and PTA.

## 2. Methods

### (a) Participants

**Figure 1.**
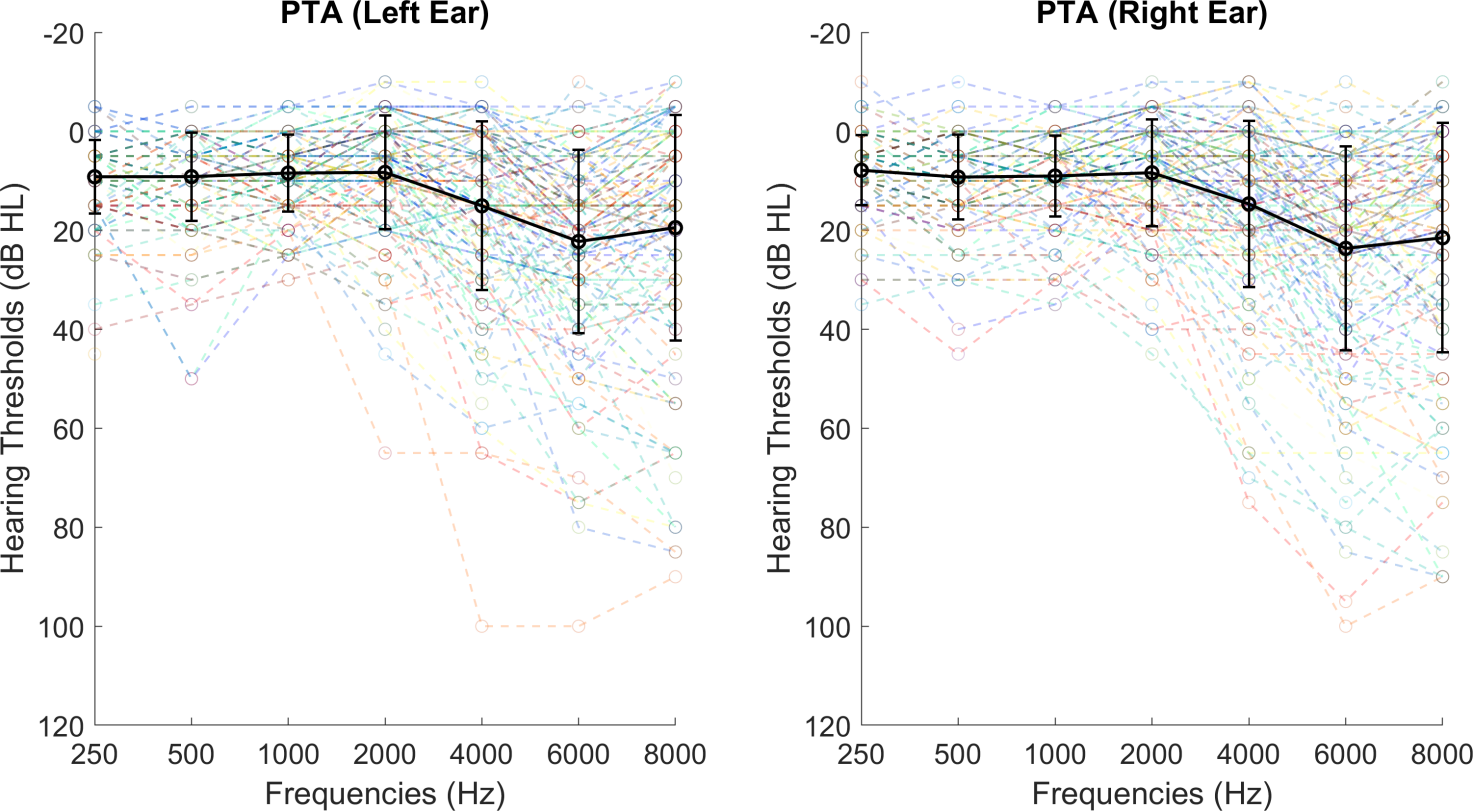
The distributions of hearing sensitivity at 250–8000 Hz for all participants' left and the right ears separately. The *x*-axis shows the frequencies measured and the *y*-axis shows hearing thresholds measured in decibels. The coloured lines with circles plot individual audiogram results and the thicker black line with circles and error bars shows the averaged group PTA. The error bars display the standard deviation.

A total of 170 participants were recruited, of whom 11 were excluded owing to data quality as per criteria described in §r4a. The final sample size used for analysis was 159. The sample had a wide range of age (mean = 45.24 years, s.d. = 18.51, range = 18–79) and hearing thresholds measured as decibels of hearing level (mean = 13.51 dB HL, s.d. = 10.05 dB HL, see figure 1 for more detailed audiogram results), with 105 female participants. All participants were neurotypical native English speakers with no history of auditory disorders, no history of speech and language disorders and who were not currently taking any psychotropic drugs. Informed consent was obtained from participants before the experiments. This study was approved by the Newcastle University Ethics Committee (46225/2023).

## 3. Stimuli and tasks

### (a) Fixed-frequency auditory figure-ground gap discrimination task

The AFG-Fixed gap discrimination test consisted of an auditory figure with temporally coherent pure-tone elements (each 50 ms duration) repeating over time. Each figure was 42 chords long with 3 figure components per chord (i.e. coherence level of 3). The figure was superimposed on an auditory ground, which is a tone cloud made of pure-tone elements (also 50 ms duration each) of randomized (or stochastic) frequencies between 180 and 7246 Hz. In each trial, two figure-ground stimuli were presented to the participants, sequentially with an interstimulus interval of 400 ms. A gap (6 chords long) was present in either figure. Although, importantly, the ground tones continued through the gap, so participants needed to have segregated the figure from the ground to perform this task. The participants were instructed to choose which of the two figure-ground stimuli contained a gap in the figure. The test used a 1-up 1-down adaptive procedure, starting at a signal-to-noise (SNR) ratio of 6 dB and varied systematically across trials. The step size started at 2 dB and went down to 0.5 dB after three reversals. Two runs were presented to each participant with different exemplars, with both runs terminating after 10 reversals. The median of the last six reversals for both runs were taken and averaged as a measure of performance. Higher SNR scores indicate worse performance.

### (b) Dynamic auditory figure-ground pattern discrimination task

**Figure 2.**
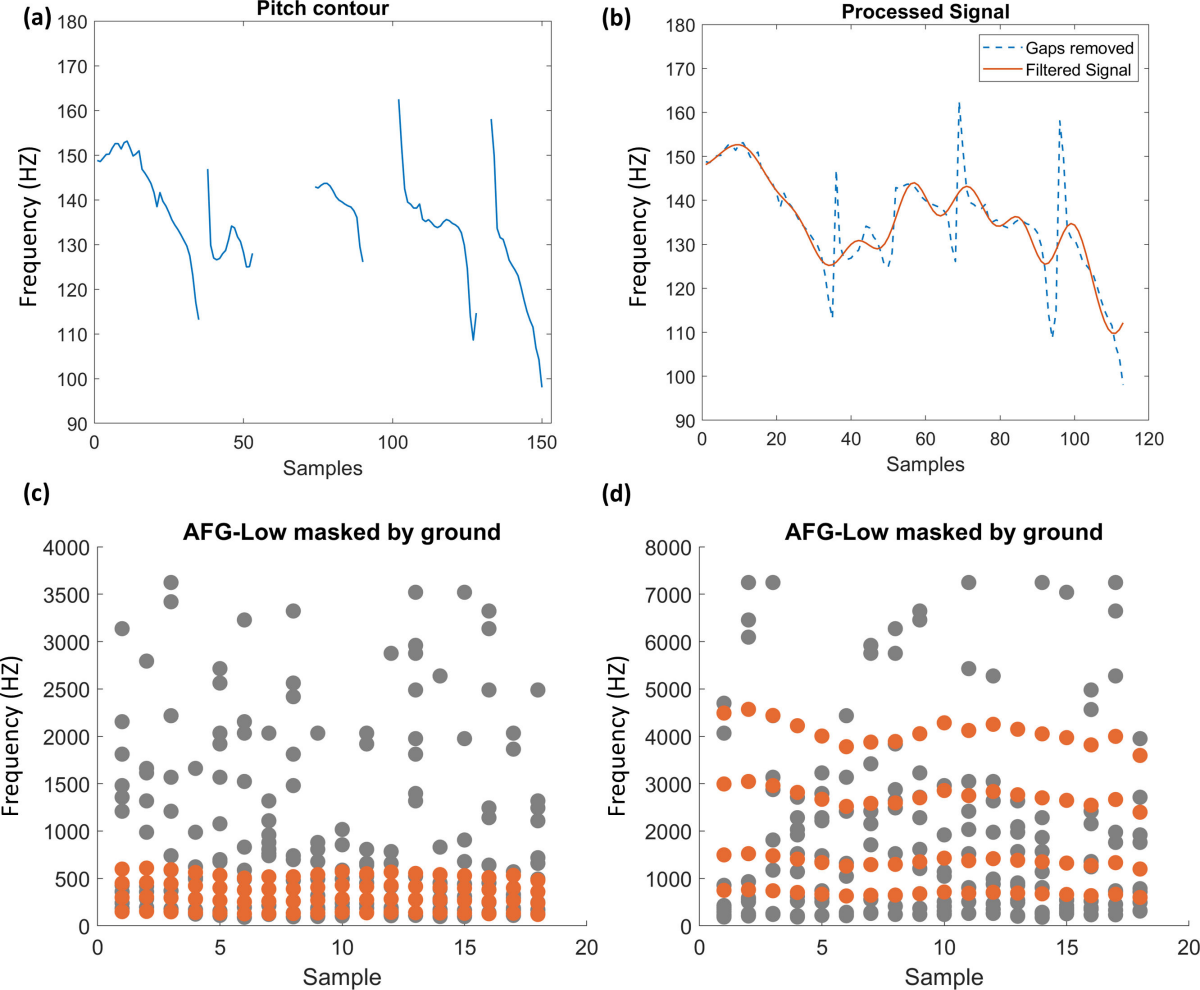
The figure shows the extraction of the pitch contour in (a,b) and the AFG stimuli with the pitch contour embedded in (c,d). Parts (a,b) show that the pitch information extracted from the sentence ‘Alan brought four small desks’. The *x*-axis plots the time in seconds and the *y*-axis shows the frequencies in Hz. Part (a) shows the raw pitch contour plotted against time. Part (b) shows the pitch trajectory after being processed. The blue line is the pitch contour with the gaps removed. The red line shows the final processed signal. The dotted plots illustrate examples of the two different types of AFG-Dynamic stimuli. Part (c) shows the lower-frequency dynamic AFG. Part (d) shows the high-frequency dynamic AFG on the right side . The *x*-axis shows the time in milliseconds and the *y*-axis shows the frequency in Hz. Figure elements are depicted in orange while ground elements are depicted in grey.

In contrast to the prototype AFG-Fixed which has a fixed-frequency pattern over time, the novel dynamic AFG contains pitch information akin to speech. The pitch contours were extracted from the English Oldenburg sentences read by a male British speaker [10], using Praat v. 6.2.09 (https://www.fon.hum.uva.nl/praat/) with a time step of 0.75/75 Hz (100 pitch values per second), and had a frequency range of 74.94–295.44 Hz (*M* = 131.59, s.d. = 15.61). The low-frequency noise (below 10 Hz) and artificial high frequencies (above 300 Hz) introduced by the Praat periodicity analysis were removed to obtain pitch trajectories (see figure 2a for an example). There are gaps in natural pitch tracks as shown in figure 2a. To avoid the participants using these gaps, the natural speech gaps and stops were first removed from the pitch contour. An example of the conjoined signal is shown in figure 2b. As demonstrated in the plot, the new signal has a general downwards trend and some sharp transitions caused by the removal of the gaps and linear interpolation. To remove the drift from individual signals, we demeaned the signal and applied detrending to the demeaned signal. Low-pass filtering (minimum-order filter with a stopband attenuation of 60 dB) with a 2000 Hz cut-off frequency was then carried out to remove the artificial spikes. The trend and the mean were then added back to the filtered signal to keep the final signal as similar to the original pitch trajectory as possible. An example of the final pitch signal is plotted in red in figure 2b.

After processing the pitch signals, the resultant frequency profiles were grouped into 50 ms-long segments by computing the average to form the figure elements. The *F*_*0*_ contour was multiplied by 2, 3 and 4 to construct the harmonic structure and these values were used to form the figure elements (figure 2c) for the AFG-Low. The tones were gated with a 10 ms raised-cosine ramp to smooth the onset and offset of the sounds. The high-frequency figure (AFG-High) retained the pitch trajectories of the low-frequency version, but the components were the fundamental frequencies multiplied by 5, 10, 20 and 30. The top frequency of each figure was checked to ensure that it did not exceed the masking frequencies. Like the AFG-Fixed stimuli, the auditory ground was composed of randomized pure-tone segments on a logarithmic scale. However, while ground tones for the AFG-High stimuli used the same range of frequencies as the AFG-Fixed (180−7246 Hz), AFG-Low stimuli used ground tones with a lower frequency range (90–3623 Hz; in other words, half of the upper and lower frequency values from the AFG-High stimuli) to achieve a better masking effect. See figure 2c,d for an illustrated example of the two types of stimuli. The duration of both AFG-Dynamic stimuli varied from 15 to 29 chords (owing to differences in sentences’ duration) randomized over the trials. Within each trial, the two stimuli were matched in length.

The two AFG-Dynamic tests (AFG-Low and AFG-High) had the same paradigm and were counterbalanced across participants. Within each test, both the figure and the ground stimuli were presented every trial, either with the same or a different figure pattern. In the case of a trial with different figure patterns, the durations of the figures were matched but the frequency elements were based on different pitch trajectories. The ground elements were tailored to different figures. The tests used a two-alternative forced-choice task, which required the participants to hold the sounds in memory and decide whether or not the second figure had the same pattern as the first one. The interstimulus (within each trial) interval was 0.2 s. A two-down one-up staircase procedure was used with a total of 22 reversals. The initial SNR was 12 dB with a step size of 2 dB, which then changed to 0.5 dB after 7 reversals. The final score was calculated by taking the median of the dB SNR of the last six reversals and a higher SNR would indicate poorer performance. The sound pairs, which were matched for the duration, and the trial orders were kept the same across participants. The same design was used for both the low-frequency and the high-frequency versions of the AFG-Dynamic test.

### (c) Speech-in-noise

Three metrics that reflected the SIN ability were used as the outcome measures, including a WiN test [27,28], a sentence-in-babble test (SiB) [10] and a subjective self-report measure (The Speech, Spatial and Qualities of Hearing Scale, ‘SSQ’) [29]. To capture the word-level perception, the B-ITCP test was used in this study, which has been demonstrated to have a close association with SiB performance [28]. Sentence-level perception is an important aspect of SIN perception and is most commonly assessed as a measure of real-life listening. The SiB test used here consists of the Oldenburg sentences and babble noise and has been used previously as an assessment of SIN perception [10].

### (d) Word-in-noise test

The WiN test was the British Iowa Test of Consonant Perception (B-ITCP) adapted from the Iowa Test of Consonant Perception [27]. Target speech sounds were monosyllabic words with a consonant–vowel–consonant (CVC) or CVCC structure. The test used British accents including one female and one male voice. The babble noise was an 8-talker babble, presented at a −2 dB SNR. The onset of the auditory target was between 0.5 and 1.0 s, randomly positioned from the babble onset. The babble segment of each trial was randomly selected from a 15 s babble stimulus. The length of the words varied from 0.304 to 0.757 s (mean: 0.508 s, s.d.: 0.086 s). Participants were asked to choose the word they heard out of a list of four words displayed on the screen balanced for difficulty and phonetic contrasts. Per cent correct across trials was taken as the outcome measure for the WiN test. This is the only test that was scored differently as it was not based on an SNR threshold but the proportion of correct responses, and a higher score for the WiN test indicates better performance.

### (e) Sentence-in-babble test

The target sentences were English Oldenburg sentences consisting of five words following a structure of Name–Verb–Number–Adjective–Noun (e.g. ‘Alan brought four small desks’). The sentences were masked by 16-talker babble. The target sentences appeared 500 ms after babble onset and ended 500 ms before babble offset. Participants were shown a 5 10 matrix on the screen, where each word in the sentence had 10 options. A correct response was counted if all five words were chosen correctly. The test used a one-down one-up adaptive paradigm with the starting SNR at 0 dB. The total number of reversals was 10 and the step size began at 2 dB and decreased to 0.5 dB after 3 reversals. The task had two runs interleaved. The target sentences were different in each run. The final score was calculated by averaging the dB SNRs of the last six reversals across the two runs. A lower score in this test indicates better performance.

### (f) Speech, spatial and qualities of hearing scale

The subjective self-report SIN ability was assessed using the SSQ [29]. Two of the questions were removed from the shortened speech-hearing questionnaire due to their ambiguity. See electronic supplementary material, appendix 1 for the full list of questions used in this questionnaire. Each item has a score from 0 to 10 with the higher score indicating more difficulty in hearing.

### (g) Procedure

An audiometry test was carried out, followed by the five computer tasks, which were presented in a fixed order for all participants, except that the order of the AFG-High and the AFG-Low tests was counterbalanced across participants. The tasks were presented in the following order: (i) SiB, (ii) AFG-Dynamic test (AFG High or AFG-Low, determined by counterbalancing across participants), (iii) SSQ, (iv) WiN, (v) second version of the AFG-Dynamic test (AFG-High or AFG-Low, whichever they had not already completed), (vi) AFG-Fixed. Participants were asked to sit in front of a computer monitor (Dell Inc.) used to present the tasks. The auditory stimuli were presented through headphones (Sennheiser HD 380 Pro) linked to a sound card (RME FireFace UC).

## 4. Data analysis

### (a) Test of correlation for AFG-Dynamic

The outcome measures of SiB and the AFG tests were the medians of the last six reversals. The performance was considered stable if the performance differences of the last six reversals were smaller than ±5 dB. Participants who did not show stable performance were excluded from the final analysis. Bivariate correlations and hierarchical regressions were carried out to explore the relationship between AFG-Dynamic and SIN tests. Tests of normality (Shapiro–Wilk) showed that the datasets were not normally distributed, so Spearman’s rho was used to examine the hypotheses regarding the relationships between the three speech measures with AFG-Dynamic (low and high version), AFG-Fixed, PTA and age. Holm–Bonferroni correction was applied to correct for multiple comparisons based on 7 × 7 pairs of comparison. As linear regression is a more tolerant measure for non-normality, stepwise regression was conducted to check if there were predictive relationships between SIN and AFG as well as specifying the variance explained by individual predictors. These tests were performed using SPSS 29 (https://www.ibm.com/support/pages/downloading-ibm-spss-statistics-29) and visualized with MATLAB R2021a (https://www.mathworks.com/help/matlab/release-notes-R2021a.html).

### (b) Modelling the inter-relations of predictors of SIN

To account for the inter-relationships of the indicator variables, SEM was conducted using the lavaan (v. 0.6-15) package (https://lavaan.ugent.be/history/dot6.html) in R (v. 4.2.1) (https://cran.r-project.org/bin/windows/base/old/4.2.1/). Maximum-likelihood estimation was used with non-normality correction based on the Satorra–Bentler scaled test statistic. Robust measures were reported in this study [30,31].

**Figure 3.**
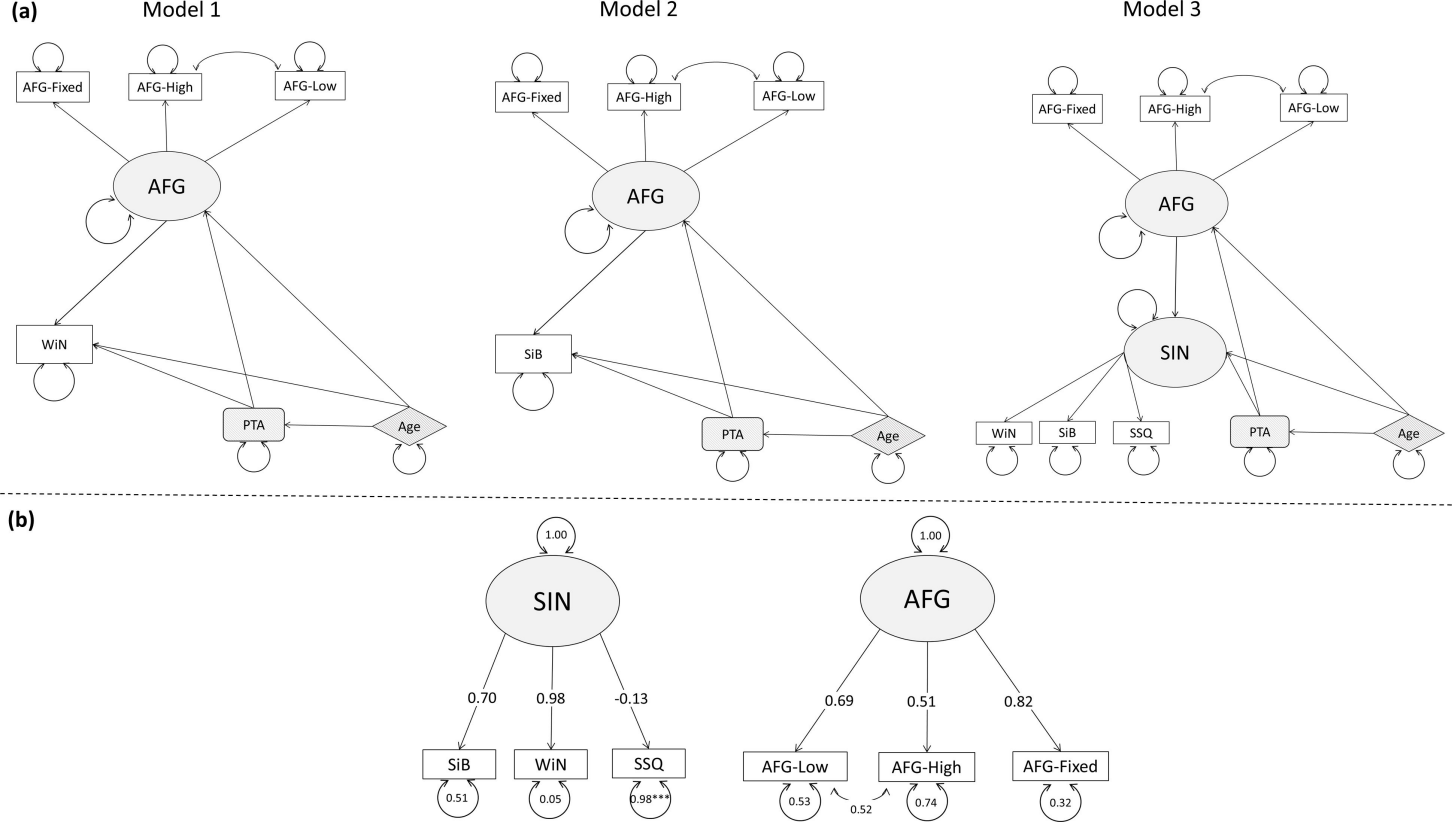
Panel (a) shows the conceptual models of WiN (model 1), SiB perception (model 2) and SIN with combined word and sentence perception (model 3). The shaded ovals represent latent variables, the rectangles represent observed variables under latent structures, the rectangles with rounded edges represent observed variables not under any latent construct and the diamonds with striped shading represent exogenous variables. The arrowed circle of each variable represents the error (the size of the circle is not proportional to the error). The indicators have arrows pointing to them from the latent variables. Arrows point from the exogenous variables towards the latent variables to suggest the former's causal effect on the latent variables. Panel (b) shows the CFA of SIN and AFG. Shaded ovals represent the latent variables, rectangular boxes are the indicators and the circles associated with each variable are the residuals. Latent variables are connected to their indicators through arrows pointing to the indicators. The error for SIN and AFG is 1 as they are not subject to any causal influences in this limited model.

Initial conceptual models (models 1 and 2) are illustrated in figure 3a. Models 1 and 2 were devised to explore word- and sentence-level SIN analysis separately. Model 3 illustrates a combined model of all three SIN measures. In all three models, the SIN measures were predicted by AFG indicated by the AFG-Fixed, and two AFG-Dynamic measures. PTA and age also predicted SIN as well as AFG. To decide the latent variable structure, a confirmatory factor analysis (CFA) was used to examine the measurement quality on a subset of the data (101 participants) before conducting the final analysis. Figure 3b demonstrates the CFA models of the two latent constructs in the three models: SIN and AFG. While there are no rules of thumb defining the acceptable thresholds of a factor loading, SSQ as a measure of functional hearing should predict a large variance of SIN tests similar to the other two SIN indicators. The SSQ, however, had a visibly weak connection to SIN and thus was removed from further analysis. All three AFG indicators seemed to be acceptable to be entered to the final model.

The results of this analysis were used to guide the selection of scaling variables [32]. Scaling variables are used to assign scales to latent variables, which is essential when identifying a model. The method used in lavaan is the Fixed Marker (FM) scaling that fixes the loading of the chosen scaling variable to 1 [33]. The choice of the scaling indicators can determine the means and variances of the latent variables thus impacting the magnitude of the unstandardized regression path estimates [34] but it is less likely to affect the model fits based on the maximum-likelihood estimation [32]. The standardized estimates are reported in this study. The path coefficients (abbreviated as *β*) can be interpreted as one s.d. increase of variable A leads to a β s.d. increase of variable B while all other relevant connections are held constant. The residual or measurement error of the indicators represents variance unexplained by the measure ‘due to random measurement error, or score unreliability’ [35].

The WiN measure was chosen as the scaling variable based on its high path coefficient connecting to the SIN latent variable. WiN was the only test measured by percentage, which resulted in a difference in scale of the outcome compared with the other tests. This was rescaled via *z-*scoring (removing the mean and dividing the results by the s.d. of the original scores of WiN). Importantly, contrary to the measures assessed with SNR, a higher score of WiN indicated better performance. This means one s.d. increase from the mean in WiN would lead to a β s.d. decrease in SIN. However, since WiN was used as the scaling variable, the SIN latent variable took the scale of WiN, while SiB instead showed a negative path coefficient. The different interpretation of SNR- and per cent correct-based scoring would further influence other factors connecting to SIN. To avoid confusion and simplify results interpretation, the WiN results were multiplied by −1 so a higher score would indicate worse performance.

AFG-High, AFG-Low and AFG-Fixed are the indicators of the latent variable AFG. Similarly, the AFG-Fixed was chosen as the scaling variable owing to its close connection with the AFG latent variable. AFG-High and AFG-Low were created with similar parameters except for the frequency range and should tap into very similar mechanisms, hence they covary. The three SEM models further consisted of age and PTA as exogenous variables, which were both configured to predict SIN and AFG.

The model fit was evaluated by a set of criteria [35,36]. These include the Bentler comparative fit index (CFI) and Tucker–Lewis Index (TLI), the root mean square error of approximation (RMSEA), the standardized root mean squared residual (SRMR) and the *χ*^2^ test. Both RMSEA and SRMR are absolute measures of the estimated discrepancy between the predicted and observed models. The SRMR is a measure of the mean absolute correlation residual measuring the differences between the original correlations (observed) and the implied correlations by the model. RMSEA ≤ 0.06 and SRMR ≤ 0.08 have been suggested to indicate a close model fit [36]. RMSEA up to 0.10 is considered a fair fit, but above 0.10 is generally unacceptable [37]. CFI and TLI, on the other hand, are incremental indices that reflect the relative improvement of the model fit compared with a baseline model [35]. TLI is non-normed so it can fall outside the 0−1 range, whereas CFI is normed, but the cut-off for both of them is above 0.95 for a good fit [36]. The *χ*^2^ result was also reported [35]. The null hypothesis for the *χ*^2^ test is that the predicted model reflects the true data perfectly. Thus, a non-significant *χ*^2^ would indicate a good model fit. Finally, bootstrap analysis was performed by randomly extracting 95% of samples (*n* = 100 times) to provide a distribution of the estimated RMSEA. Confidence intervals (CIs) of the path estimates for all three models were calculated based on the bootstrapped estimates (CI = mean ± margin of error). The data and SEM analysis scripts are freely available online [48].

## 5. Results

The descriptive statistics are reported in table 1.

**Table 1.** The mean and s.d. of the participants’ performance on the five computer tasks.

	mean	s.d.
SiB	−0.880	2.114
WiN	0.673	0.107
AFG-Low	8.991	10.897
AFG-High	7.252	10.447
AFG-Fixed	−14.542	8.200

### (a) Relationships between SIN measures and AFG-Dynamic

**Figure 4.**
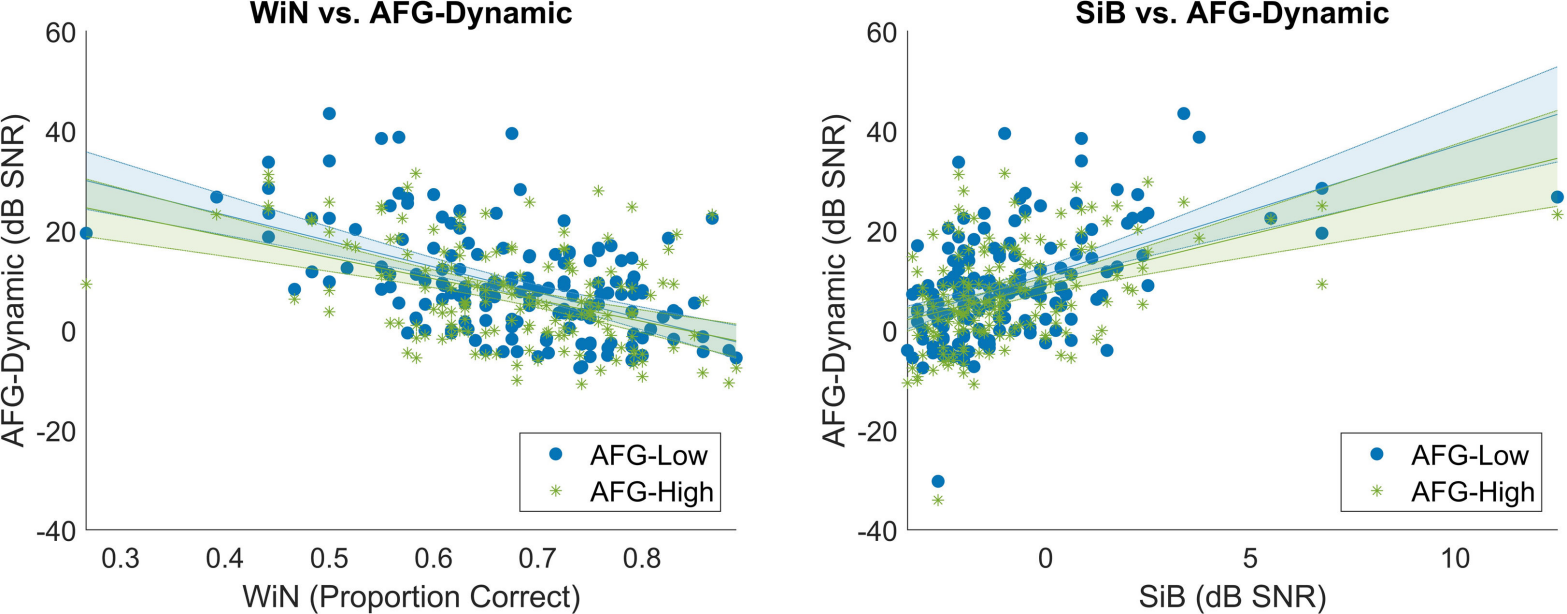
Scatterplots of AFG-Dynamic and SIN measures. The lines of best fit were plotted as straight lines in the figure with shaded error bars. The *x*-axis for the left plot showed the WiN results as proportion correct (number of correct answers divided by the overall number of trials) and the *x*-axis for the right one showed SiB thresholds measured in dB SNR. The *y* axes of the were the two AFG tasks measured in dB SNR.

Both the sentence- and word-level SIN tests showed moderate to strong correlations with the dynamic AFG measures (figure 4). After correction, all *p*-values remained highly significant. SSQ, however, did not show any significant correlation with other speech measures (*p* > 0.34) and was removed from further analysis. The *r*-values and corrected *p*-values of corrections are summarized in table 2.

**Table 2.** This table summariz the *r*-values of the correlation test. The *p*-values are reported as asterisks: two asterisks represent *p* < 0.01, and three asterisks represent *p* < 0.001.

	WiN	PTA	age	AFG-High	AFG-Low	AFG-Fixed
SiN	−0.56***	0.57***	0.50***	0.42***	0.47***	0.57***
WiN	—	−0.67***	−0.73***	−0.39***	−0.47***	−0.61***
PTA	—	—	0.72***	0.24**	0.36***	0.59***
age	—	—	—	0.28**	0.35***	0.55***

The hierarchical regression predicting SiB performance gave three significant predictors, revealing that PTA, AFG-Low and AFG-Fixed performance significantly predicted SiB performance (*F*_3, 155_ = 39.879, *p* < 0.001). The model accounted for 43.56% of the variance in SiB. Table 3 specifies the variance explained by the significant predictors. For the WiN model, four predictors were significant: age, PTA, AFG-Low and AFG-Fixed (*F*_4, 154_ = 62.560, *p* < 0.001). The model accounted for 61.90% of the variance in WiN. Table 3 specifies the variance explained by each predictor. For SiB, PTA was the best predictor explaining about 31% of the model with the AFG-Low adding 9.9% to the model, whereas, for WiN, age seemed to be the strongest predictor. Of the significant predictors, AFG-Fixed added the least variance to both SiB and WiN, which was about 1–2% after accounting for the other variables.

**Table 3.** This table displays the adjusted *R*² values and *p*-values of models including an increasing number of predictors that add significant variance to the models predicting either SiB or WiN.

SiB	standardized coefficients beta	adj *R*²	*p*	WiN	standardized coefficients beta	adj *R*²	*p*
PTA	0.368	0.314	<0.001	age	−0.409	0.498	<0.001
+AFG-Low	0.253	0.413	<0.001	+AFG-Low	−0.217	0.580	<0.001
+AFG-Fixed	0.197	0.436	0.015	+PTA	−0.218	0.609	0.003
+AFG-High	0.126	—	0.125	+AFG-Fixed	−0.134	0.619	0.049
+Age	0.074	—	0.400	+AFG-High	−0.121	—	0.075

## 6. Structural equation model of SIN, AFG, hearing and age

**Figure 5.**
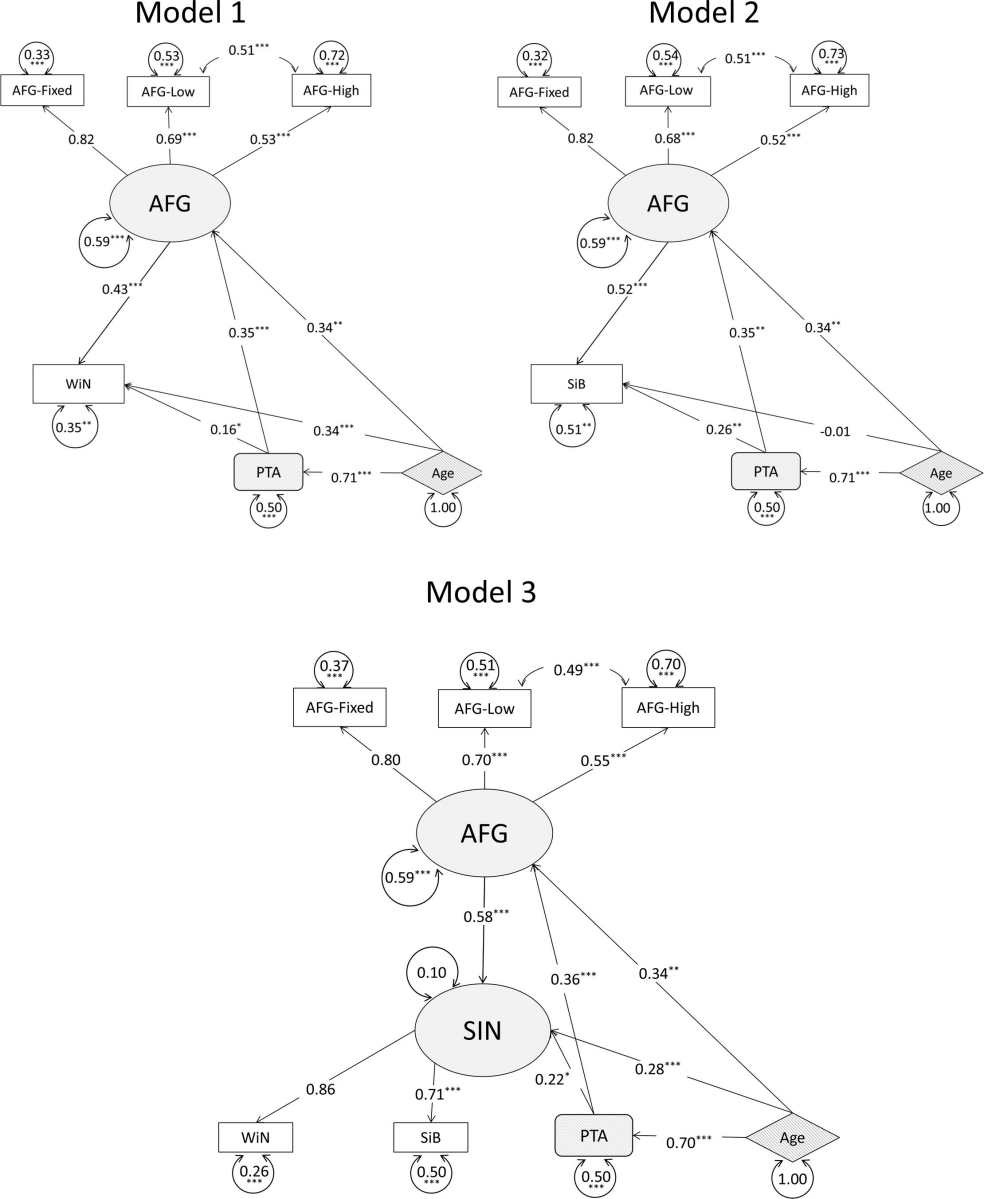
Models 1 and 2 are presented with either the WiN measure or the SiB measures as the dependent variable, model 3 had WiN and SiB combined as the dependent variable. All indicator variables were plotted in rectangles. The oval shape represents the latent variable (AFG) in both models, and the exogenous variables are plotted in a diamond shape. The latent variable measured by indicators has arrows pointing towards the indicators. Otherwise, the arrows point from the variable that causes a change in another one. The path coefficients were marked by numbers and error terms are marked by an encircled number. The significance level was marked by asterisks. Three asterisks represent *p* < 0.001, two represent *p* < 0.01 and one represents *p* < 0.05. Note that while AFG-Fixed in all three models and WiN in model 3 were not marked with asterisks, it was not because they failed to predict the latent variables but because the scaling variables were not estimated in the SEM. The scaling variables dominate the latent constructs.

The fit indices for the three models are shown in table 4, and path coefficients are plotted in figure 5. The confidence interval of the path estimates of the three models were summarized in electronic supplementary material, appendix II. All fit indices for models 1 and 2 were within our criteria. The path coefficients in model 1 were all significant. Model 2 had mostly significant paths with a non-significant one of age to SiB. Model 3 followed the conceptual model structure shown in figure 3 but had the path connecting SSQ to SIN removed as it was not significant. This model met most of the set criteria for an excellent model fit except for the RMSEA. RMSEA incorporates model complexity and models with smaller degrees of freedom tend to obtain a poorer RMSEA [38]. This pattern of results is similar to that obtained for models 1 and 2, which also had excellent fit based on most of the indicators but poorer than expected RMSEA. However, as the combined results of other indicators all showed that the model fits the data very well, we deem that this model is acceptable. All three models were accepted based on the fit criteria. The bootstrapped RMSEA of the three models overlapped over 18%, so there was no significant difference between their model fit (See electronic supplementary material, figure S1).

**Table 4.** Fit indices for models 1, 2 and 3. Adjusted *R*² also reported in the last row per model.

fit index	model 1	model 2	model 3
*χ* ^2^	9.547 (*p* = 0.067)	7.910 (*p* = 0.122)	20.617 (*p* = 0.009)
RMSEA	0.079	0.065	0.092
CFI	0.990	0.992	0.978
TLI	0.969	0.975	0.949
SRMR	0.028	0.029	0.036
Adj *R*²	0.617	0.435	0.889

Models 1 and 2 reported similar adjusted *R*² as the regression results. As expected, in both models, all three AFG indicators showed significant contributions to the AFG indicator, and the AFG-High and AFG-Low shared significant covariance. AFG-Fixed contributed to AFG with the largest path coefficient (|*β*| = 0.82) followed by the two dynamic AFG measures. The latent AFG variable predicted WiN and SiB significantly, with the largest variance compared with PTA and age in both models. PTA had a significant but smaller contribution to each SIN measure. Age only has a significant direct impact on WiN and not on SiB.

Model 3 explained 89% of the SIN variance (combined word and sentence measure). Similar to models 1 and 2, AFG explained the largest variance of the latent SIN variable (*β* = 0.58) in model 3 (figure 5), compared with age and PTA. Both SiB and WiN showed significant contributions to the latent SIN variable, but WiN had a numerically greater contribution (|*β*| = 0.86 for WiN compared with |*β*| = 0.71 for SiB). Age was the second largest predictor of SIN, after AFG, and PTA had a smaller (but nevertheless significant) direct impact on SIN. However, both PTA and age had a significant indirect impact on SIN through AFG.

## 7. Discussion

### (a) Predicting SIN perception with dynamic AFG in the linear models

This study showed a moderate to strong correlation between all AFG measures and SIN, both on the word and the sentence level. The correlation between AFG-Fixed and SiB reported previously (*r* = 0.32) [10] was replicated and showed a larger effect (*r* = 0.57) possibly owing to a larger sample size. The low-frequency AFG came out as a significant predictor of WiN and SiB, explaining the largest variance in both models after accounting for demographic factors (age or PTA). It is unexpected that even for the WiN model the AFG-Low explained more variance than the static AFG. The dynamic AFG was designed to carry the fundamental frequency patterns and so should better predict sentence-level sound segregation than word-level. AFG-Fixed, on the other hand, had no frequency change over time, which was considered more similar to WiN perception. Based on the regression results, however, it seems that adding the speech pitch pattern to the AFG stimuli only improved its predictive power of SIN in general, not specific to sentence-level perception. This general improvement could be the reason the AFG-Fixed did not explain a higher portion of the variance of SIN as well. Considering that AFG-Low combined both the mechanism of segregating static figures from ground (by employing the figure’s temporal coherence feature) with the speech-like frequency pattern to aid SIN perception, it is reasonable to see higher variance obtained by AFG-Low in a linear model.

One possible explanation for the relationship between AFG-Low and both word and sentence-level SIN is its harmonic structure. AFG-Fixed differed from AFG-Dynamic in two ways: it is both static and inharmonic. Some of the AFG-Fixed stimuli might contain near-harmonic figures by chance, but most of the stimuli were inharmonic, which elicit weaker pitch perception [39]. Pitch plays an important role in SIN perception [16,40–42], the mechanism of which was reviewed by Oxenham [12]. This includes not only its strong association with the stress contour of a whole sentence but also other linguistic features such as phonemes in words. The pitch information embedded in the AFG-Low can help with differentiating the envelope fluctuations of the target sound from the background sound, which is key for speech intelligibility. Thus, the stronger pitch strength could be the reason why AFG-Low, while sharing the same basic principles with the static AFG, predicts SiB or WiN better.

he high-frequency dynamic AFG had a numerically weaker correlation as was hypothesized and did not explain additional variance in WiN or SiB after accounting for other tasks. This could be because AFG-High shared a high covariance with AFG-Low owing to the similar parameters used for these two tests. The AFG-Low more closely resembles the speech stimuli used in this study with its frequency range being closely configured to the pitch range of speech formants, which might be the reason why AFG-Low outperformed AFG-High in predicting SIN.

The SSQ measure did not correlate with either of the speech measures, which was not a unique finding [43,44]. This could be because the shorter SSQ version does not have enough sensitivity to capture SIN perception as only a few questions were related to speech comprehension in human speech noise.

### (b) Predicting SIN perception in a multivariate model

The linear regression models displayed the core contribution of the new dynamic AFG measure as well as the static measure. However, the stepwise procedure did not account for the interaction between variables. The SEM model provided a more comprehensive picture of the experiment that went beyond ranking the important predictors of SIN measures.

First, models 1 and 2 showed that all three AFG predictors have an impact on the SIN performance. This means that when accounting for the interaction and covariance shared between the indicators, all of the AFG predictors should be considered a necessary part of the AFG analysis. Interestingly, while the linear measures showed a tighter relationship between AFG-Low and WiN/SiB, AFG-Fixed in the SEM models contributes the most to the AFG latent variable. This suggests that as the ‘prototype’ AFG, the static AFG that assesses people’s ability to pick up the temporally coherent figure from the tone cloud, is still the core of the AFG analysis process. Combined with the regression results, it shows that the dynamic pitch pattern does add an important aspect to AFG and should be used in combination with AFG-Fixed. Based on their individual predictive value in the regression results, AFG-Low should be a better test to measure SIN ability compared with AFG-Fixed in a linear model, when using both measures is not possible.

The combined AFG measures explained the largest variance (43 and 52%) of both speech measures in models 1 and 2, compared with age and PTA. This also differs from the linear regression results, where PTA or age was found to be the greatest predictor. This difference suggests that AFG tasks have greater predictive power in combination as various ways to assess scene segregation, whereas each AFG task individually assesses slightly different abilities that are weaker individually than the influence of the demographic factors. The lower path coefficient of PTA compared with AFG indicates that the ability to process speech (either single-word utterances or sentences) in a noisy environment directly relies more on segregating auditory streams and tracking the pattern of the target sounds over time than simply picking up acoustic signals as measured by PTA. However, PTA also had a mediation effect on WiN/SiB through a large path coefficient to AFG. This means that in addition to a relatively small direct impact on SIN perception, deteriorated peripheral hearing could alter functional hearing through modifying central sound processing, which is consistent with our hypothesis.

A mediation effect was also evident with the age-driven impact on SIN perception. Age led to around 70% SD change in PTA in this study, meaning the PTA variance was largely dominated by age-related hearing loss. Age also decreased central sound processing measured by AFG here by 34%, consistent with previous results [10]. However, while age showed a significant correlation with SiB, it did not modify SiB performance directly in the SEM model, which is consistent with the regression results. WiN is a harder task for people who are older or with higher hearing thresholds. This is because less in the way of compensatory mechanisms can be employed for hearing a short word compared with a sentence that contains a legitimate syntactic structure. While normal ageing can result in deteriorated hearing sensitivity and the perception of other acoustic properties (fine structure or harmonicity), language perception skills are generally preserved [45].

The interaction among predictors in model 3 did not change much after combining the WiN and SiB into one latent variable. WiN and SiB had a similar level of contribution to the SIN latent factor and the small residual term of SIN suggests that WiN and SiB together provide a holistic measurement of SIN, with a small effect of unmeasured sources of unique variance on the latent variable. It is important to highlight, however, that combining the measures into a latent variable could hide the different effects of other predictors such as age, like in models 1 and 2.

### (c) Limitations and future direction

The sample size of the current study might have caused the fit to be suboptimal. There is no golden rule in terms of determining an appropriate sample size for SEM. Researchers have suggested a variety of standards based on the number of observations (*N*) per statistical estimates (*q*) ranging from 20 : 1 to 5 : 1 depending on the complexity of the model [35,46] or an absolute sample size of 250 if using the Satorra–Bentler scaled method [36]. The current study has around 8:1 *N*:*q*, which is sufficient to find a good solution to meet the convergence criteria, but not optimal. Further studies are needed to validate the model with a larger sample size.

This study also focused mainly on individuals without symptomatic hearing impairment. The new dynamic measures will need to be tested on different populations such as hearing impaired or patients with cochlear implants, to see whether the results can be replicated with people who struggle with SIN perception. Indeed, recent data suggest that AFG-fixed does predict SIN performance in CI users [47], so it is plausible that AFG-dynamic in CI users may explain even further variance. This then can potentially be used for clinical practice to assess patients’ dynamic sound segregation. Future research can also incorporate other aspects of SIN perception (e.g. subcortical sound analysis, language ability) and cognitive measures (general intelligence, auditory working memory) to test if the effect of AFG on SIN holds when accounting for these other factors.

Finally, the pattern discrimination task design of the dynamic figure-ground was inherently more challenging than gap detection. While this would ensure figure-tracking and improve its predictive power of SIN perception, it would also involve a higher working memory load, making the task less perceptual. This should be taken into account when choosing which figure-ground task to use. The performance of AFG-Fixed shown in this study might be impacted by fatigue, although this effect should be relatively small. The AFG-Dynamic and AFG-Fixed were always run after the SIN tests: one AFG-Dynamic was run after 25 min of SiB testing, and AFG-Fixed followed 20 min of WiN and AFG-Dynamic testing. This design was to ensure the best performance on the two speech tasks, but future studies should consider counterbalancing the task orders to minimize fatigue.

In conclusion, these data show that an adequate model of SIN perception needs to account for age, peripheral auditory function and measures of grouping that we have previously demonstrated to have a brain basis. We introduced new measures of central grouping in this work that incorporate harmonicity and a pitch trajectory taken from natural speech. These measures have improved the prediction of speech in noise in the multivariate model.

## Data Availability

The data and analysis script that support the findings of this study are openly available in OSF at [48]. Supplementary material is available online [49].
